# Effects of dietary copper on organ indexes, tissular Cu, Zn and Fe deposition and fur quality of growing-furring male mink (*Mustela vison*)

**DOI:** 10.1186/s40781-015-0040-x

**Published:** 2015-02-25

**Authors:** Xuezhuang Wu, Xiuhua Gao, Fuhe Yang

**Affiliations:** Feed Research Institute, Chinese Academy of Agricultural Sciences, Beijing, 100081 China; State Key Laboratory for Molecular Biology of Special Economic Animals, Institute of Economic Animal and Plant Science, Chinese Academy of Agricultural Sciences, Changchun, 130112 China

**Keywords:** Mink, Copper, Organ indexes, Fur quality

## Abstract

The objectives of this study were to study the effects of different levels of dietary copper on organ indexes, tissular Cu, Zn and Fe deposition and fur quality of mink in the growing-furring periods. One hundred and five standard dark male mink were randomly assigned to seven groups with the following dietary treatments: basal diet with no supplemental Cu (Control); basal diet supplemented with either 6, 12, 24, 48, 96 and 192 mg/kg Cu from copper sulphate, respectively. The colour intensity scores displayed a linear trend (P = 0.057). The spleen Cu concentrations responded in a linear (P < 0.05) fashion with increasing level of Cu, but copper supplementation did not affect speen concentrations of Fe or Zn. Supplemental dose of Cu linearly increased (P < 0.05) liver Cu and Fe concentrations but did not alter (P > 0.10) liver Zn. Our results indicate that Cu plays an important role in the pigmentation in growing-furring mink, and supplemental dietary Cu in growing-furring mink improve hair colour, and copper has limited effects on liver mineral deposition.

## Background

Copper is a key trace mineral in mink nutrition in terms of its role in hemoglobin formation and normal pigmentation of fur. Copper, as an essential trace element, is required for plant, animal, and human health as well as for fur production of mink. Copper is a constituent of several metalloenzymes such as cytochrome oxidase, which functions in cellular respiration, and lysyl oxidase, important in connective tissue formation [1,2]. Copper is also a constituent of tyrosinase, an enzyme involved in melanin (the black pigment of skin, hair, and fur) [3-5]. The recommended level for copper in the mink diet is 4.5-6.0 mg/kg (NRC) [6]. However, earlier studies by Aulerich and Ringer [7] indicated superior weight gains in male kits but not in female kits fed supplemental copper at the 50 mg/kg level. In two other studies, Aulerich et al. [8] and Bush et al. [9] it was noted that darker fur color occurred in male kits but not female kits with supplemental copper at the 200 mg/kg level. Studies at Cornell University with mink on purified diets suggested that 20 mg Cu/kg DM was adequate for growth and fur development [10].

Although copper has been found to play an important role in the nutrition and fur development of mink, the effects of copper supplementation on organ indexes, tissular Cu, Zn and Fe deposition and fur quality of mink has never been reported. Furthermore, the optimal dietary level of Cu in mink has not been established. Therefore, the objectives of the present study were to investigate the effects of dietary copper level on organ indexes, tissular Cu, Zn and Fe deposition and fur quality of mink in the growing-furring period. The optimum dietary copper requirement for mink was also estimated.

## Methods

The animal protocol for this experiment was approved by the Animal Care Committee of the Institute of Special Economic Animal and Plant Science of the Chinese Academy of Agricultural Sciences (44.02。N, 126.15。E). Animals were maintained and processed in accordance with the CAAS Guide for the Care and Use of Laboratory Animals.

### Animals, diets, management, and experimental design

One hundred and five standard dark male mink were randomly assigned to seven groups with the following dietary treatments: basal diet with no supplemental Cu (Control); basal diet supplemented with either 6(Cu6), 12(Cu12), 24(Cu24), 48(Cu48), 96(Cu96) and 192(Cu192) mg/kg Cu from copper sulphate, respectively. The basal diet mainly consisted of corn, fish meal, meat bone meal and soybean oil, with no Cu supplementation. The experiment period lasted for 98 days. The composition and chemical analysis of the basal diet are shown in Table 1.

**Table 1:** **Ingredient and chemical composition of basal diet**

**Items**	**Contents**
Ingredient [g/kg diet]	
Extruded corn	312
Soybean meal	60
Corn gluten meal	80
Fish meal	180
Meat and bone meal	180
Cheese meal	30
Soybean oil	120
Feather meal	10
Blood meal	10
Premix^a^	10
Lysine	3
Methionine	3
NaCl	2
Total	1000
Chemical composition [g/kg]	
Dry matter [g/kg]	976.0
Crude protein [g/kg DM]	330.5
Crude fat [g/kg DM]	167.2
Crude carbohydrate [g/kg DM]	424.1
Ash [g/kg DM]	60.2
Contents of mineral elementsa	
Calcium [g/kg]	32.2
Phosphorus [g/kg]	22.0
Copper [mg/kg]	7.6
Zinc [mg/kg]	43.6
Iron [mg/kg]	203.4
Contents of amino acida [mg/kg]	
Lysine [g/kg]	16.9
Methionine [g/kg]	9.3
Cysteine [g/kg]	3.6
Metabolizable energy^b^ [MJ/kg DM]	20.3
% from protein	30.6
% from fat	32.7
% from carbohydrates	36.7

^a^Contained the following per kg of premix:vitamin A, 1 000 000 IU; vitamin D3, 200 000 IU; vitamin E, 6 000 IU; vitamin B1,600 mg;vitamin B2, 800 mg; vitamin B6, 300 mg;vitamin B12 10 mg; vitamin K3,100 mg; vitamin C, 40 000 mg;niacin acid, 4 000 mg;pantothenic acid, 1 200 mg;biotin, 20 mg;folic acid, 80 mg;choline, 30 000 mg; Fe, 8 200 mg; Mn, 1 200 mg; Zn, 5 200 mg; I, 50 mg; Se, 20 mg; Co, 50 mg.

^b^Metabolizable energy was calculated according to Hansen et al. [11].

The animals were housed individually in open-sided sheds in mink growing cages (60 cm long × 40 cm wide × 50 cm high) with additional attached nest boxes (30 cm long × 40 cm wide × 30 cm high). All animals were vaccinated with distemper and canine parvovirus before the study started. Animals had access to ad libitum clean drinking water (contained less than 0.01 mg Cu/L by analysis). Animal had access to feed and tap water ad libitum throughout the study. Measured amount of treatment diets were offered twice daily at 08:00 and 16:00.

### Chemical analysis

The chemical composition of the diets were analyzed by standard methods. Dry matter was determined by drying feed samples at 105°C to constant weight. The Cu, Fe and Zn content of feed and organs samples [12] were estimated in an air–acetylene flame on an atomic absorption spectrophotometer (Shimadzu Scientific Instruments, Kyoto, Japan).

### Slaughter traits

Eight minks selected randomly from each group were pelted in accordance with normal farming practice. Mink were killed by gas (CO2) in a killing box, 20–30 mink may be placed in there, depending on box-size, according to the Welfare of Animals Kept for Fur Production. The abdominal cavity was opened and various organs obtained. The liver, spleen, heart, and kidney were weighed to determine the relative organ weight. Organ index was determined using a method described by Lu et al. [13]. Organ index (g/kg) = weight of organ/empty-body weight.

Fur properties evaluated on dried skins. Leather thickness was measured 15 cm anterior from the tail root along the middle of the back with a modified micrometer. Overall quality was visually assessed on a scale ranging from 1 to 10 (best), and silkiness was visually assessed on a scale ranging from 1 to 4 (most silky and glossy), Colour intensity [1 (light)–4 (dark)] was measured by the optic technique of the Finnish Fur Sales. Underfur density was examined in skin biopsies. The skin biopsies were prepared as described by Rasmussen and Damgaard [14] for histology.

#### Statistic analysis

Data were analyzed using the general linear models (GLM) Procedure of SAS [15]. The following model was used:

$$ \mathrm{Y}\mathrm{i}\mathrm{j}=\mu +\mathrm{d}\mathrm{i}+\varepsilon \mathrm{i}\mathrm{j}, $$

Where Yij is the observation; μ is the general mean; di is the effect of Cu level (i = 1,…,7); εij is the random error.

Tukey tests were used to detect statistical significance between treatment groups. Linear and quadratic effects due to copper level were determined. Significant differences were accepted if P ≤ 0.05.

## Results

### Organ indexes

There were no differences in the relative weights of the spleen, heartand kidney among the seven treatments (P > 0.05, Table 2). The relative weight of the liver slightly increases with the increase of dietary copper.

**Table 2:** **Effects of copper supplementation on organ indexes in growing-furring male mink**

**Treatments**		**Liver (g/kg)**	**Spleen (g/kg)**	**Heart (g/kg)**	**Kidney (g/kg)**
Control		31.28	3.78	6.5	3.55
Cu6		31.47	3.86	6.2	3.75
Cu12		31.47	4.00	5.8	4.58
Cu24		32.41	4.04	6.5	3.44
Cu48		33.33	3.81	7.4	3.61
Cu96		33.10	4.09	5.9	4.19
Cu192		34.63	3.92	6.2	3.52
SEM		0.32	0.11	0.15	0.13
p value	Linear	0.078	0.615	0.681	0.535
	Quad	0.400	0.647	0.561	0.442

Notes: Data are expressed as Least Squares Means with pooled SEM; n = 8 per treatment.

### Quality of skins

The overall fur quality responded in a quadratic (P < 0.05) fashion with increasing level of Cu; best overall fur quality were seen in the Cu24 group (Table 3). The colour intensity scores displayed a linear trend (P = 0.057) with the maximum response in the Cu192 group, but copper supplementation did not affect leather thickness and underfur hair density.

**Table 3:** **Effects of dietary copper on quality of skins in growing-furring mink**

**Treatments**		**Overall quality (score)**	**Colour intensity (score)**	**Silkiness (score)**	**Underfur hairs per mm2 skin**	**Leather thickness (mm)**
Control		7.72	2.30	2.85	241.2	0.77
Cu6		7.95	2.32	3.10	245.2	0.73
Cu12		7.96	3.00	2.91	239.0	0.69
Cu24		9.13	3.17	3.19	247.4	0.73
Cu48		8.66	3.02	3.32	250.9	0.84
Cu96		8.78	2.98	2.66	247.7	0.89
Cu192		8.67	3.17	3.00	247.0	0.89
SEM		0.11	0.10	0.10	1.49	0.01
p value	Linear	0.026	0.057	0.738	0.279	0.982
	Quad	0.012	0.186	0.894	0.157	0.350

Notes: Data are expressed as Least Squares Means with pooled SEM; n = 8 per treatment.

### Tissular Cu, Zn and Fe deposition

Copper level had no effect (P > 0.10) on kidney concentrations of Cu, Fe, or Zn (Table 4), indicating that final levels of these elements were similar across treatments. The spleen Cu concentrations responded in a linear (P < 0.05) fashion with increasing level of Cu, but copper supplementation did not affect liver concentrations of Fe or Zn. Supplemental dose of Cu linearly increased (P < 0.05) liver Cu and Fe concentrations but did not alter (P > 0.10) liver Zn. The heart concentrations of Cu slightly increases (P = 0.094) with the increase of dietary copper.

**Table 4:** **Effect of Cu supplementation on tissular Cu, Zn and Fe deposition in growing-furring mink**

**Treatments**		**Liver (DM basis, mg/kg)**			**Spleen (DM basis, mg/kg)**			**Heart (DM basis, mg/kg)**			**Kidney (DM basis, mg/kg)**		
		**Cu**	**Zn**	**Fe**	**Cu**	**Zn**	**Fe**	**Cu**	**Zn**	**Fe**	**Cu**	**Zn**	**Fe**
Control		16.25	81.17	473.8	12.98	96.24	1039.4	13.26	69.89	244.2	21.43	68.11	343.8
Cu6		19.00	97.33	445.1	15.57	94.22	1014.8	10.36	67.70	229.2	20.87	67.12	336.4
Cu12		32.36	93.28	447.1	15.85	95.44	1022.6	11.00	68.86	234.7	20.16	63.62	331.3
Cu24		67.13	96.92	445.8	17.68	97.98	1073.0	11.88	70.69	242.3	21.39	69.16	345.5
Cu48		66.44	81.55	416.3	18.73	95.48	998.4	12.86	68.89	240.0	21.34	68.06	342.9
Cu96		73.47	87.98	414.8	22.11	95.29	920.0	14.01	68.75	241.9	21.42	68.54	344.6
Cu192		96.50	95.77	409.7	26.48	95.41	942.3	15.36	68.84	239.9	20.87	67.59	357.4
SEM		4.67	1.67	9.31	0.79	0.76	14.8	0.32	0.54	2.21	0.34	1.22	5.62
p value	Linear	0.001	0.473	0.002	0.001	0.335	0.335	0.094	0.952	0.512	0.609	0.638	0.888
	Quad	0.002	0.212	0.412	0.179	0.459	0.459	0.689	0.994	0.589	0.602	0.679	0.883

Means with different letters within a row differ significantly (P < 0.05); data are expressed as Least Squares Means with pooled SEM; n = 8 per treatment.

## Discussion

We found that the liver weights of the pelted males were not affected by supplemental dietary copper. Results of the present study are consistent with observations of Aulerich et al. [8].

In the present study, hair colour of the mink fed with the various concentrations of Cu was evaluated because fur colour is an important characteristic in dark mink. Copper, as a constituent of tyrosinase, plays a key role in a normal pigmentation of fur in mink [4,16-18]. Previous study [8,19] has reported that relatively high levels (100–200 mg/kg) of supplemental Cu may have a beneficial effect on intensifying hair colour of dark mink. Bush et al. [9] also demonstrated that darker fur colour occurred in male kits but not female kits with supplemental copper at the 200 mg/kg level. One of the clinical signs of Cu deficiency in mink is hypochromotrichia and some mink farmers have reported feeding supplemental dietary Cu to intensify fur colour in dark mink [20].

Because the liver is one of the main organs involved in the storage and metabolism of Cu, liver Cu concentration might be considered indicative of an animal’s Cu status. Copper concentrations in the liver were in accordance with normal for most adult non-ruminant animals (15 to 30 mg Cu/kg DM), and were within the broad range reported by Fisher [21] and Aulerich et al. [8]. These data suggest that mink may be similar to sheep and swine in that liver Cu stores increase in proportion to dietary Cu intake [22]. Stejskal et al. [23] found that copper was concentrated in the livers and tended to increase with age for mink. Heart copper concentrations also can increase in mink fed high dietary copper, but to a lesser extent than liver. Arredondo [24] reported that copper deficiency generates cellular iron deficiency. It is noteworthy that feeding supplemental dietary copper to decrease Fe concentrations in the liver. Ceruloplasmin oxidizes Fe^2+^ prior to transferrin binding [25]. The absence of ceruloplasmin does not produce marked changes in Cu metabolism. It does, however, produce a gradual accumulation of Fe in the liver and other tissues [26]. During severe Cu deficiency, Fe transport within the body is adversely affected, and Fe tends to accumulate in many tissues. Generally, Cu deficiency is accompanied by a hypochromic microcytic anemia similar to that produced by Fe deficiency.

## Conclusions

Our results indicate that Cu plays an important role in the pigmentation in growing-furring mink, and supplemental dietary Cu in growing-furring mink improve hair colour. Data also indicate that copper has limited effects on liver mineral deposition.
